# Polyphenolic-Rich Compounds From *Dillenia pentagyna* (Roxb.) Attenuates the Doxorubicin-Induced Cardiotoxicity: A High-Frequency Ultrasonography Assisted Approach

**DOI:** 10.3389/fphar.2021.624706

**Published:** 2021-05-17

**Authors:** Kalyani Tene, M. Kalyan Kumar, G. Basveshwar, P. Eswara Rao, G. Jagadeesh Kumar, Pramod kumar, Deepak B. Pemmaraju, U. S. N. Murty, Ranadeep Gogoi, V. G. M. Naidu

**Affiliations:** ^1^ Department of Biotechnology, National Institute of Pharmaceutical Education and Research- Guwahati, Assam, India; ^2^ Department of Pharmacology and Toxicology, National Institute of Pharmaceutical Education and Research-Guwahati, Assam, India; ^3^ Department of Pharmaceutical Analysis, National Institute of Pharmaceutical Education and Research Guwahati, Assam, India; ^4^ Biological Sciences and Technology Department, CSIR-North-East Institute of Science and Technology, Assam, India

**Keywords:** doxorubicin, oxidative stress, cardioprotection, ultrasonography, *dillenia* pentagyna roxb

## Abstract

Cardiovascular complications are the foremost concern in patients undergoing anticancer therapy*.* There is an unmet need to address the problems arising from the drug-induced toxicity for the long-term benefit of the patients undergoing chemotherapy. Alternative medicines are gaining their prosperity in addressing the various drug-induced organ toxicity. *Dillenia pentagyna* Roxb (DP) is an ethnomedicinal plant rich in flavonoids and phenolic contents. In India & Nepal, DP is a common ingredient of traditional medicines used to treat multiple ailments like inflammation, cancer, and diabetes. However, its protective role against doxorubicin (Dox) induced cardiotoxicity remains unexplored. Herein, we investigated the potential effects of various extracts/fractions obtained from the DP’s bark against Dox-induced cardiotoxicity, both *in-vitro* and *in-vivo*. The anti-oxidant content of the extracts/fractions was evaluated by using DPPH, ABTS and FRAP chemical assays. The results indicated that the hydroalcoholic (HA) extract of DP has intense anti-oxidant potential. Further fractionation of DP revealed that the phenolic-rich fraction (F1) has a high anti-oxidant potential. The protective effect of extract/fraction was also investigated in the H9c2 cell line following the Dox-induced cardiotoxicity model. We observed that the pre-treatment of extract/fraction in cardiomyocytes had exhibited increased cell viability. Fluorescence-based chemical assays indicated a decreased ROS levels in the treated groups in comparison to the Dox control group. The effect of DP was evaluated further in balb/c mice by the Dox-induced cardiotoxicity model. Non-invasive techniques like high-frequency ultrasonography and electrocardiogram revealed that the mice pre-treated with DP had improved cardiac functionality (left ventricular ejection fraction and stroke volume) and normalized the electrocardiograms compared to the Dox control group. Further, biochemical analysis with the cardiac tissues revealed that the cytoprotective proteins like HO-1, SOD-2, and Nrf-2 were elevated in the DP treated groups compared to the Dox control group. Overall, our results suggested that the bioactive extract/fractions of DP helped alleviate the Dox-induced cardiotoxicity. LC-QTOF-ESI-MS analysis of DP and F1 indicated that polyphenolic anti-oxidant compounds like gallic acid, syringic acid, and sinapic acid could be responsible for the potent -cardioprotective effect. Future understanding of the pharmacokinetics and pharmacodynamic parameters can help translate from the bench to the bedside.

## Introduction

*Cancer* is one of the significant public health concerns affecting nearly 18 million people, thereby leading to 9.6 million deaths worldwide (Bray et al., 2018). Current treatment strategies for cancer include targeted therapy (Ex: immunotherapy), photodynamic/thermal treatment, radiation, and chemotherapy. Among them, chemotherapeutic drugs have shown a better prognosis in reducing the tumor burden and increasing cancer patients’ overall life expectancy. Modern cancer therapies had increased the number of survivors. However, there is a surge in cardiac complications in patients undergoing various cancer therapies contributing to increased morbidity and mortality (Tan and Scherrer-Crosbie, 2014). Multiple drugs and therapies like Doxorubicin, Paclitaxel, monoclonal antibodies (Trastuzumab), and tyrosine kinase inhibitors (Imatinib), causes cardiotoxicity (Brown et al., 2015). Doxorubicin (Dox) is a widely used drug reported to induce oxidative stress-mediated necrosis in the cardiomyocytes (Xu et al., 2020). A single regime of Dox at a dose of 60–75 mg/m^2^ for every three weeks is being used in the clinical application (Rugo et al., 2016). Increased dosage of Dox leads to its accumulation in cardiolipin (a phospholipid located in the inner mitochondrial membrane) of cardiomyocytes leading to the disruption of the electron transport chain, ultimately leading to the generation of ROS (Huang et al., 2015). Reports indicated that the Dox also reduces the functional capacity of the Nrf-2 gene, which is a critical regulator of the redox system (Wang et al., 2019). This affects the reduction of anti-oxidant enzymes like glutathione (GSH), and catalase (Peoples et al., 2019). Few Nrf-2 activators have been developed (Cuadrado et al., 2019); however, limitations like interference with drug’s oncologic efficacy and low bioavailability had hindered their complete translation. Therefore, creating safe and highly effective therapeutics to mitigate the toxicity associated with the chemotherapeutic drugs will benefit cancer patients.

Plants are considered a vast source of natural anti-oxidants from ancient times. Many such sources have even become part of our dietary lifestyle due to polyphenols, vitamins, mineral content. They have high nutritional value and therefore gained importance in drug discovery over time. *Dillenia pentagyna* Roxb (DP) belongs to the family Dilleniaceae, commonly known as Nepali elephant apple, is a highly valued medicinal plant distributed in Asian countries. Fruits of it are edible and are cooked as a dish by the tribal people of North-East and central parts of India. The plant’s bark and fruits have been traditionally used for treating inflammatory diseases, chest pain, and cancer (Sharma et al., 2001). The plant’s seed and bark are also used against cancer by the Koch-Rajbanshi tribes of northeastern states in India (Yadav and Srivastava, 2014). Fruits of the plant possess flavonoid glycosides like naringenin 7-galactosyl and dihedral quercetin 5- galactoside (Saiful Yazan and Armania, 2014).

Preliminary *in-vitro* studies on the bark showed anti-oxidant content due to the presence of lupeol, betulin, and betulinic acids. Despite the therapeutic properties, the plant or its parts were not scientifically explored for beneficial effects in managing drug-induced oxidative stress-mediated diseases/toxicity. In this regard, the current study intends to evaluate *Dillenia pentagyna* Roxb (DP) extract/purified fractions ameliorative effect against Dox-induced cardiotoxicity in both *in-vitro* and *in-vivo* studies. High-frequency ultrasonography assisted real-time monitoring of cardiac functional parameters was attempted. To the best of our knowledge, we are the first to understand the potential of DP against Dox-induced cardiotoxicity using high-frequency ultrasonography (HFUS).

## Materials and Methods

### Chemicals

Doxorubicin (Dox), DPPH, ABTS, NBT, DTNB, and Lactate dehydrogenase (LDH) assay kit were purchased from Sigma Chemicals (St. Louis, MO, United States). Ascorbic acid was purchased from Hi-media. Creatine Kinase-MB (CK-MB) was procured from Accurex (Mumbai, India). Normal goat serum, Alexa Flour 488 goat anti-rabbit, and Hoescht’s 33,258 were procured from Invitrogen. All primary (Nrf-2, SOD-2, Keap-1, and HO-1) and secondary antibodies used in this study were obtained from Cell Signalling Technology (Beverly, Massachusetts United States). LC-MS grade water and acetonitrile were purchased from M/s JT Baker, United States. Ammonium acetate and formic acid were brought from M/s Sigma Aldrich, United States. All the chemicals were used as such without any modification and derivatization.

### Cell Lines and Maintenance

Rat cardiomyocytes (H9c2 cell line) were procured from NCCS, Pune and colon cancer cell line (HCT-116) was procured from ATCC, United States. The cells were cultured in DMEM, and RPMI medium respectively enriched with fetal bovine serum (10% v/v), L-Glutamine (1%), and anti-anti (antibiotic and antimycotic) (100°U/ml) and was maintained at 37°C in a humidified air containing 5% CO_2_ in a sterile condition.

### Animals

25–30°g male Balb/c mice were used to evaluate the bioactive DP’s cardio-protective activity (hydro-alcoholic). Animals were procured from Palamur Biosciences (Hyderabad, India). All experiments were performed by following the Committee for the Purpose of Control and Supervision of Experiments on Animals (CPCSEA), India. The study was approved by the Institutional Animal Ethics Committee (IAEC), Guwahati, India (NIPS/NIPER/18/029). The animals have been housed in individually ventilated cages (IVC) under standard conditions (temperature 23 ± 1°C, 12 h light/dark cycle). Acute oral toxicity study was performed for the maximum dose of 2000 mg/kg for seven days as per Organization for Economic Co-operation and Development (OECD) guidelines.

### Plant Material Collection

The bark of *Dillenia pentagyna* Roxb. was collected from Auxiguri village, Baksa district (91° 20.47′ E and 26° 39.49′ N), Assam in the month of April. The plant was authenticated from Bodoland University Herbarium, Kokrajhar, with accession number (BUH 0000142).

### Preparation of Bioactive Extract

Bark was shade dried at room temperature and pulverized into fine powder through a grinding mill (MF 10 B S000, IKA, Germany). The fine powder was extracted with successive solvents with low polarity to high polarity index. The powder was first defatted with the hexane and macerated twice with each solvent for 24 h in an incubator shaker. The solvents were followed as chloroform (CH), ethyl acetate (EA), ethanol (EOH), and hydro-alcohol (HA) (1:1). The extracts were filtered using Whatman filter paper Grade-1 and subjected to dryness using a rotary evaporator (IKA RV 10, Germany).

## *In-vitro* Anti-oxidant Assays

All the extracts were tested for preliminary anti-oxidant potential by using the following assays.

### DPPH Radical Scavenging Assay

All the extracts and fractions of DP were tested for their free radical scavenging properties using a Spectramax I3X (Molecular Devices, United States). 20 μl aliquot of different concentrations of the extracts, fractions (1,000, 500, 250, 125, 62.5 and 31.25 μg/ml) and the respective standard (Ascorbic acid 200, 100, 50, 25 and 12.5 μg/ml) were added to 200 μl of DPPH in methanol (0.2 mM). After 20 min of incubation, the absorbance of each solution was read at 517 nm. Results were expressed in percentage free radical scavenging activity (% RSA) with reference to ascorbic acid (Sridhar and Charles, 2019), and IC_50_ values were calculated.

### ABTS Radical Scavenging Assay

All the extracts and fractions of DP were tested to scavenge the cation radicals. To 20 μl of various concentrations of the test samples (1,000, 500, 250, 125, 62.5 and 31.25 μg/ml) or standard 200, 100, 50, 25 and 12.5 μg/ml), 200 μL of ABTS solution was added and incubated for 20 min. The resulting absorbance of these solutions was measured spectrophotometrically at 734 nm using Spectramax I3X. Analyses were done in percentage radical scavenging activity (% RSA). Ascorbic acid was employed as the reference standard (Abramovič et al., 2017), and IC_50_ values were calculated.

### FRAP Assay

All the extracts and fractions of DP were tested for their ability to reduce Fe^+3^ to Fe^+2,^ which can be observed from light green to blue color at an absorbance of 700 nm. Ascorbic acid was used as a reference standard. 0.2 ml of each test sample of different dilutions (1,000, 500, 250, 125 and 62.5 μg/ml) was added to 0.2 ml of PBS and 0.2 ml of 1% ferric chloride. The reaction was incubated for 20 min at 50 °C, and the reaction was stopped by adding 0.2 ml of 10% trichloroacetic acid, 0.5 ml of distilled water, and 0.2 ml of 0.1% ferric chloride. Absorbance was measured against a blank solution containing all the reagents except the test samples or standard. The value of FRAP was expressed in mg ascorbic acid equivalent (AAE)/Gram of extract (Müller et al., 2011).

### Total Phenolic Content

Total phenolic content of all the extracts and fractions of DP were estimated by the Folin–Ciocalteu method. Bioactive fractions 100 μl (1 mg/ml) and mixed thoroughly with 0.2 ml of Folin–Ciocalteu followed by adding 0.75 ml of sodium carbonate solution 75% (w/v) and incubated for 30 min and read at 765 nm. Results were expressed as mg of Gallic acid Equivalent (GA)/Gram of extract (Alizadeh Behbahani and Shahidi, 2019).

## Method Development for Separation of the Active Constituents

Flash Chromatography was used for the separation of active components. Solvent optimization was carried out initially using thin-layer chromatography (TLC) on Silica gel 60 F_254_ (Merck, Germany) A range of solvents with different polarity indexes was used, and finally, methanol and water (9:1 ratio) was found to be optimal to separate maximum constituents. Later, the crude extract was loaded onto the flash chromatography apparatus (Biotage Isolera one, Sweden), and the chemical components were eluted using the mobile gradient phase by reverse phase chromatography. The solvents used are methanol (A) and water (B) (A: B) 100–90% with a flow rate of 20 ml/min using a Sfar C18 30gD Duo 30um cartridge. Three fractions (named Fraction F1, F2, and F3) were collected and evaluated for *in-vitro* anti-oxidant potential, and further biological activity screening was performed.

### *In-vitro* Cardioprotective Studies

#### Cell Viability

Cells were seeded at the density of 1 × 10^4^/well and incubated for 12 h. Bioactive fractions were treated with different concentrations (6.25, 12.5, 25, and 50 μg/ml) for 24 h. MTT assay assessed cell viability followed by treatment with bioactive extract/fraction with or without Dox treatment. 0.5 mg/ml MTT was added to the cells and incubated for 4 h. DMSO was added to dissolve the insoluble formazan crystals and read the plate at 570 nm by Spectramax I3X (Van Meerloo et al., 2011).

#### Measurement of ROS and Superoxide Anion Radicals

Cells were plated 1 × 10^6^/well and pre-treated with bioactive fractions for 2 h, and Dox 40 µM was given for 24 h. Cellular ROS generation was determined qualitatively by CM-H2DCFDA and evaluated using a fluorescence microscope (EVOS FL Auto two, Invitrogen). The fluorescein intensity was quantitatively assessed by a Flow cytometer (Life Technologies, Thermo fisher scientific, Singapore). Cellular Superoxide radical generation was defined by Mitosox red. H9c2 cells were subjected to the same treatment as ROS and superoxide radical was evaluated by a confocal microscope (Leica, Germany). Results were expressed as mean fluorescence intensity (MFI).

#### Bioactive Fraction/s and Dox on Cancer Cell Line

Cells were seeded at the density of 5 × 10^3^ cells/well and incubated for 12 h. Bio-active fractions were treated with different concentrations 3.125.6.25, 12.5, 25 and 50 μg/ml for 24 h. Extracts were treated along with Dox (2.5 μM) for 24 h, and an MTT reagent was used to assess the cell viability. 0.5 mg/ml. MTT was added to the cells and incubated for 4 h. DMSO was added to dissolve the insoluble formazan crystals, and the plate was read at 570 nm by Spectramax I3X (Molecular Devices, United States of America).

### *In-vivo* Studies

#### Experimental Design

Mice were divided into four groups, each group consisting of six animals. Group 1: Normal saline control. Group 2: disease control, received an intraperitoneal injection of Dox 2.5 mg/kg six times in two weeks. Group 3: Treated with the bioactive DP (100 mg/kg) per oral (p.o) daily once for fourteen days and an intraperitoneal injection of Dox 2.5 mg/kg six times in two weeks. Group 4: Given DP (200 mg/kg) (p.o) daily once for fourteen days and an i. p injection of Dox 2.5 mg/kg six times in two weeks of the study. Body weights of the mice were recorded at periodic intervals during the study to evaluate the treatment’s effect (Rugo et al., 2016).

#### Electrocardiographic Recording (ECG) and Analysis

The ECG was performed for different groups on day 14^th^ (end of the experiment). Briefly, mice were anesthetized with 4% isoflurane and maintained anesthesia with 1–2% isoflurane and followed by insertion of electrodes through a needle in the right hind limb, right forelimb, left forelimb. The ECG was recorded for each mice and data was collected by AD Instrument, Australia. T wave elevation, *p* duration, and ST heights were analyzed using Lab Chart 8 software (AD Instrument, Australia) (Sheibani et al., 2020).

#### Analysis of the Cardiac Parameters by High-Frequency Ultrasonography Imaging

The animals underwent imaging to evaluate the cardiac functional parameters. The animals were anesthetized with 4% isoflurane in an anesthesia chamber and placed on a heating pad, and anesthesia was maintained using 1.5% isoflurane by a nasal mask throughout the experiment. Echocardiography was performed using a high-frequency (30 MHz) small animal imaging system (Vevo LAZR-X 3100, FUJIFILM Visual Sonics). The transducer used was MX400 with a mouse small cardiology application and the parasternal long-axis view (Gangrade et al., 2020; Tene et al., 2020). Cardiac functions like cardiac output, fractional shortening, and ejection fraction were calculated from B mode where left ventricular region of the heart was focused in a two-dimensional manner. Similarly, systolic volume, diastolic volume, left ventricular mass, left ventricular posterior wall thickness systolic (LVPW; s) left ventricular posterior wall thickness diastolic (LVPW; d) were measured for each animal from the M-mode image. Measurements were performed using digital image analysis software (Vevo Lab 3.1.1).

#### Assessment of Anti-Oxidant Enzymes

Serum was collected and stored at −20°C. CK-MB, LDH assays were measured with corresponding assay kits from Accurex and Sigma. Heart tissue was homogenized with Tissue lyser II (Qiagen, Germany) using 0.05M PBS pH 7.4 and centrifuged at 12,000 rpm at 4°C for 10 min, and the supernatant was collected and anti-oxidant parameters GSH (Owen and Butterfield, 2010), catalase, MDA was measured. Results were expressed per mg protein (Ohkawa et al., 1979).

#### Tissue Processing

The mice were sacrificed by cervical dislocation under mild anesthesia and the heart from each mice was collected. Organs were fixed and stored in neutral 10% formalin solution for 24 h. Tissues were dehydrated in grades of alcohol and embedded in paraffin blocks. Sections (5 μm) were cut by using a microtome and placed on slides and stained with hematoxylin and eosin (H&E) staining. A light microscope analyzed the stained segments under 20x magnification (Koul et al., 2014).

#### Western Blot Analysis

The effect of DP in modulating the ROS mediated by the Dox induction was evaluated by western blot analysis of the homogenized H9c2 cells and heart tissue lysate. The cells and heart tissues were briefly collected and washed with ice-cold 1X PBS, followed by RIPA lysis buffer protein extraction (Battu et al., 2018). The supernatant was collected, and the protein concentration was determined by using the Bradford reagent at 595 nm. Equal protein concentrations were subjected to SDS-PAGE (10 and 12% Tris-Glycine gel) with a pre-stained protein molecular weight marker (Bio-Rad). The separated proteins were transferred to nitrocellulose membrane (Bio-rad) followed by blocking in 3% BSA (Hi-media) for 1 h at room temperature. Blocked membranes were incubated with the specific primary antibodies (Nrf-2, Keap-1, SOD-2, HO-1, *a*-tubulin, and *ß*-actin) overnight at 4°C, washed three times with TBST followed by incubation with secondary antibody for 1 h at room temperature. Blots were developed by using Clarity substrate solution (Peroxide solution and enhancer solution (1:1) on a Chemiluminescence 17-200255 (Fusion F_x_ Vilber 363 Lourmat) and densitometric analysis by ImageJ software (Bethesda, Maryland, United States).

### LC-MS/MS Characterization

Hydroalcoholic extract of *Dillenia pentagyna* Roxb. and its fraction F1 was used for UHPLC-QTOF-ESI-MS analysis. Dried samples were mixed with methanol and water on an equal ratio (1:1). Samples were vortexed and sonicated for uniform mixing. Samples were filtered by a 0.22 µM syringe filter to remove the foreign article and to avoid cone choking during analysis. Plant metabolites identification was performed using UHPLC-QTOF (Agilent 1,290 Infinity II Binary pump, well plate autosampler, thermostatted column compartment, and Agilent 6545XT AdvanceBio QTOF LC/MS/MS. Agilent Poroshell 120 EC C18, 2.7 µM, 2.1 × 100 mm column was used in this study with 45°C. The autosampler temperature was kept at 6°C to avoid any degradation during the analysis. The mobile phase was used in gradient mode as A and B. A is composed of 95:5: Water: Acetonitrile with 0.1% formic acid and 10 mM Ammonium Acetate, and B is composed of 95:5 Acetonitrile: Water with 0.1% formic acid and 10 mM Ammonium Acetate. The flow rate was kept at 0.4 ml per minute. Mobile phase B was changed concerning time such as 0.00 min-2% B, 3 min-2%B, 15 min-95%B, 20 min-95%B, 20.1 min-2%B, 25 min-2% B, and the stop time was 25−min. QTOF MS was run in both positive and negative mode using the Agilent Jet stream option. Source conditions were composed of gas temperature−275°C, drying gas (nitrogen)-8 L per minute, nebulizer gas (nitrogen) −35psi, sheath gas temperature −300°C, sheath gas flow-11 L per minute. Fragmentation, capillary voltage, and nozzle voltage were set at 150, 3500, and 1000 V, respectively. The mass range was kept 100–1700 m/z with one spectrum per second acquisition rate time.

#### Statistics

Mean ± SEM values were used for the expression of data unless mentioned otherwise. Statistical analyses of data were performed using the One Way ANOVA (GraphPad Software Inc., CA, United States) followed by Tukey’s post hoc test unless specified. Values of *p* < 0.05 were considered statistically significant.

## Results

### Identification and Purification of the Anti-Oxidant Fractions

**FIGURE 1:**
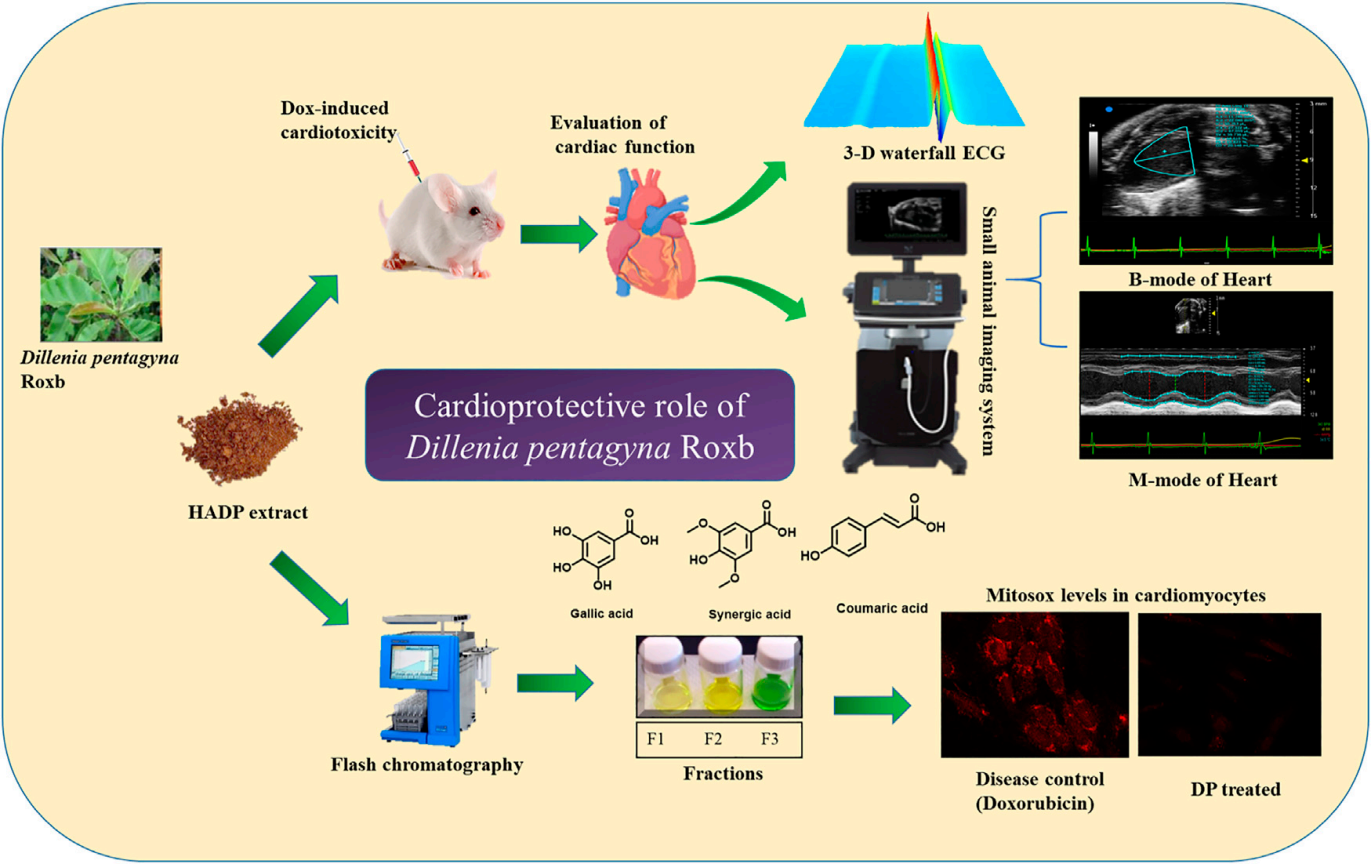
Schematic representation of the proposed study design cardioprotective mechanism by the hydroalcoholic extract of DP.

**FIGURE 2:**
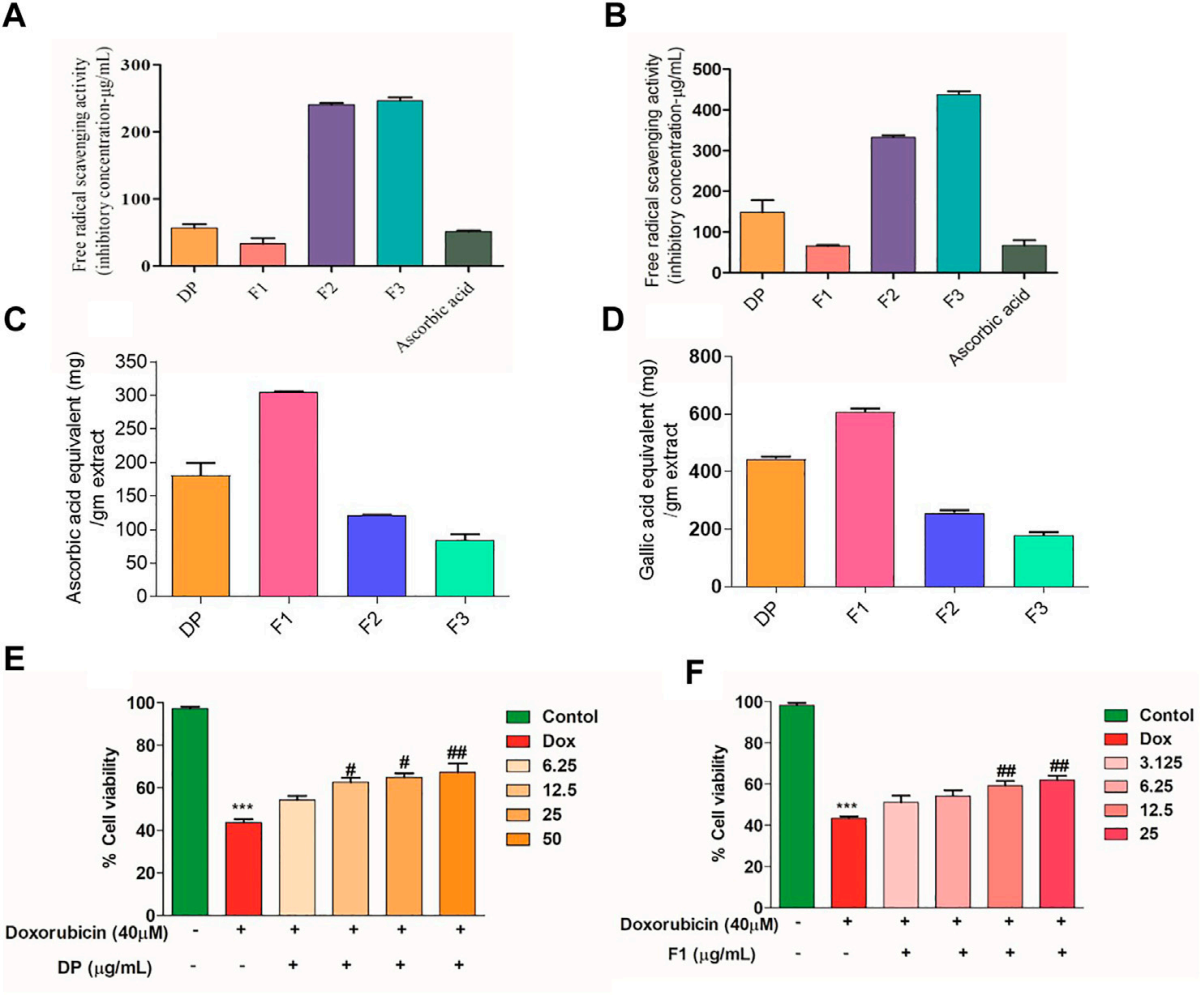
IC_50_ values of the bioactive fractions in various antioxidant assays. **(A)** ABTS and **(B)** DPPH assay compared with ascorbic acid. **(C)** FRAP assay (equivalent ascorbic acid/gm extract) **(D)** Total phenolic content (Gallic acid equivalent/gm extract). **(E, F)** Cell viability of cardiomyocytes (H9c2) pre-treatment with DP and Fraction F1 along with Dox. Values were expressed as Mean ± SEM (*n* = 3). The data were analyzed by One Way ANOVA using Graph Pad Prism. ***p < 0.001, and **p < 0.01represents Control vs Dox control. ^##^ *p* < 0.01 and ^###^ *p* < 0.001 represents Dox control vs Treatment groups.

The bark powder obtained from the *Dillenia pentagyna* Roxb (DP) was subjected to polarity guided extraction and identified that the DP hydroalcoholic extract has a potent anti-oxidant property (Supplementary Figure S1). The generalized scheme of the study is depicted in the Figure 1. The DP hydroalcoholic extract was subjected to fractionation to obtain different fractions (F1, F2, and F3) (Supplementary Figure S2). Further analysis identified that fraction F1 has shown high anti-oxidant molecule content (DPPH, ABTS, and FRAP) compared to the respective control (DP), (Figures 2A–C). The total phenolic content analysis indicated that the bioactive DP and fraction F1 have high total phenolic content, as shown in Figure 2D.

### Bioactive DP Hydroalcoholic Extract and Its Fraction F1 Alleviates the Dox-Induced Toxicity in Cardiomyocytes

Pre-treatment with the bioactive fractions (DP and F1) has shown no remarkable changes in the morphology of the H9c2 cardiomyocytes. The MTT assay result indicated that the cardiomyocytes treated with Dox exhibited 43.61 ± 1.61% of viability. In contrast, pre-treatment with the bioactive DP has alleviated the Dox’s toxic effect in H9c2 cells in a dose-dependent manner. A similar protection effect was observed when the cardiomyocytes were treated with the fraction F1, as shown in Figures 2E,F, respectively, Maximum protection was observed at the higher concentration of the DP and F1 (12.5 μg/ml). Meanwhile, bioactive extracts alone (without Dox) did not cause significant cytotoxicity (Supplementary Figures S2A,B).

### Bioactive Fraction Attenuates the Dox-Induced ROS Production *In-vitro*

**FIGURE 3:**
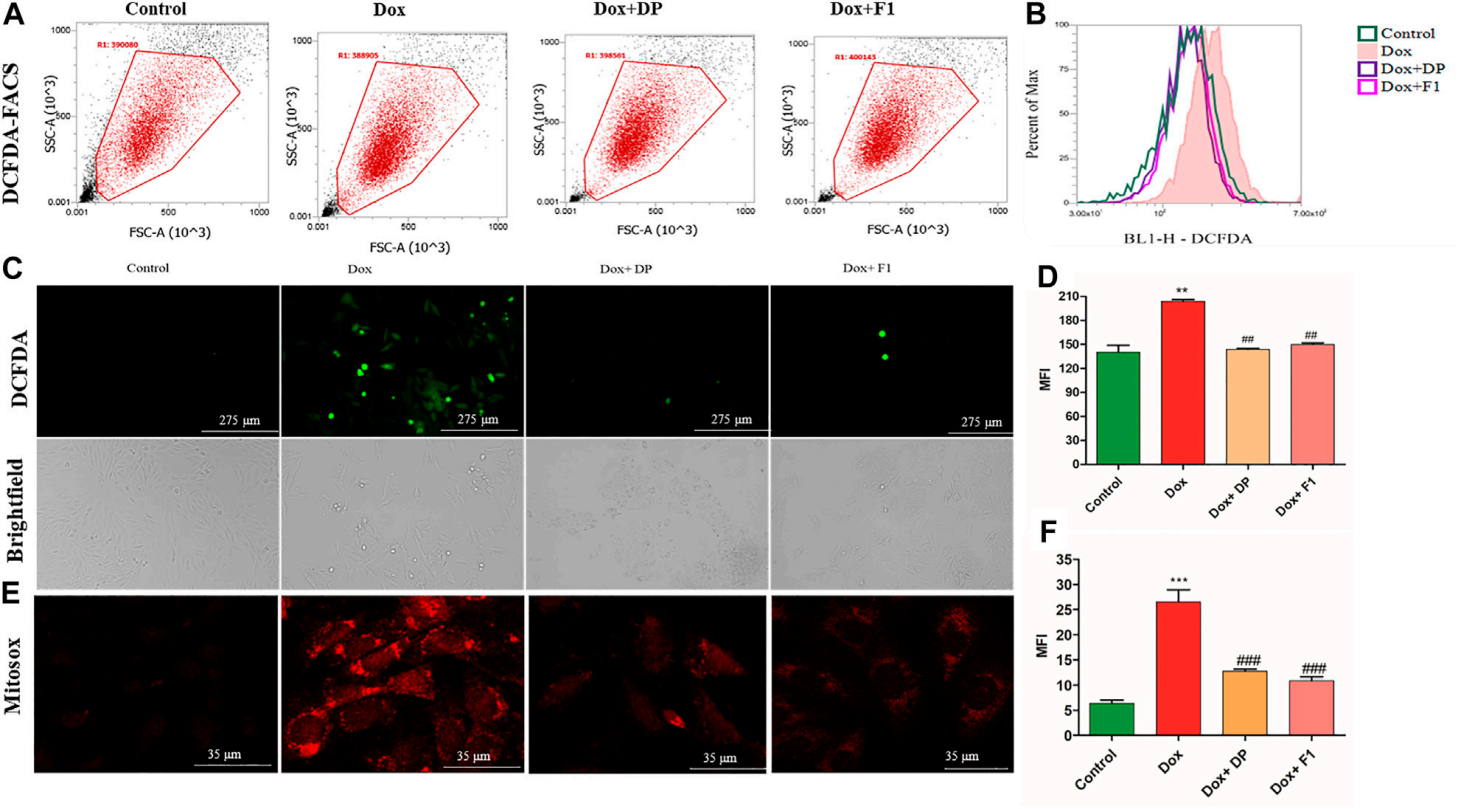
*In-vitro* evaluation of the cardioprotective effect of the DP and fraction F1. **(A)** Quantitative analysis of ROS by DCFDA using flow cytometer. **(B)** Histogram overlay of DCFDA shift **(C)** Representative brightfield and fluorescent images of the DCFDA stained cells. **(D)** Mean fluorescence intensity of DCFDA. **(E)** Representative superoxide radical generation using Mitosox staining. **(F)** Quantitative measurement of mean fluorescence intensity (MFI) of Mitosox red. The data were analyzed by One Way ANOVA using Graph Pad Prism. ***p < 0.001, and **p < 0.01represents Control vs Dox control. ^##^ *p* < 0.01 and ^###^ *p* < 0.001 represents Dox control vs Treatment groups (*n* = 3).

Dox causes cellular and mitochondrial damage by intracellular ROS generation. Hence, to evaluate the bioactive DP’s role in modulating the cellular ROS generation, we have assessed the quantitative total cellular ROS level using a fluorescent dye DCFDA by FACS. Figure 3A represents the distribution of the DCFDA stained cell populations. As evidenced in Figure 3B, there is a right shift of the cell population in the Dox control group, indicating an overall increase in ROS level compared to the control group. When pre-treated with the bioactive DP and its fraction F1, the cardiomyocytes have shown ROS levels equivalent to the control group. The fluorescent microscope images also indicated a high fluorescence in the Dox group and basal amounts of intracellular ROS in the cardiomyocytes pre-treated with the bioactive DP and fraction F1, as shown in Figure 3C. The quantified bar graph represents their mean fluorescence intensities (MFI) in Figure 3D. The Dox-treated group of cardiomyocytes has higher mean fluorescence intensity (MFI:204 ± 2.0) than the control group (140 ± 9.0).

Whereas the pre-treatment with DP (12.5 μg/ml) and F1 (12.5 μg/ml) had significantly reduced the ROS levels (143.5 ± 1.5 and 150 ± 2.0, respectively). Mitochondrial superoxide was assessed using Mitosox red by a confocal microscope. The mean fluorescent intensity in Dox control remarkably increased compared with the control group, where pre-treatment with bioactive fractions attenuated the superoxide generation in mitochondria in Figures 3E,F. The results indicated that the cardiomyocytes, upon pre-treatment with the bioactive DP and its fraction F1 even after the induction with Dox, have successfully alleviated the increased ROS levels.

### Bioactive Fraction Did Not Compromise the Anticancer Activity of Dox

Both the DP hydroalcoholic extract and fraction F1 were co-treated with Dox (2.5 μM) in a cancer cell line (HCT116) to understand if they have any interference. We observed that the cancer cell death was similar in the groups treated with Dox alone and Dox combined with the DP hydroalcoholic extract and fraction (Supplementary Figures S3C,D). Overall, the results indicate that the DP hydroalcoholic extract and fraction F1 had not compromised the Dox anticancer property. However, considering the scalability, cost of production, and effort to green technology contribution, we have opted for the DP hydro alcoholic extract for the *in-vivo* studies.

### *In-vivo* Cardioprotective Role of the Bioactive DP

Acute toxicity studies indicated that the bioactive DP was safe at a dose of 2000 mg/kg in mice, where no toxicity was observed for 14 days a single administration. Therefore, by following the OECD guidelines 425, we have chosen 1/20^th^ (i.e., 100 mg/kg) and 1/10^th^ (200 mg/kg) of the maximum tolerable dose for further studies.

### Bioactive DP Hydroalcoholic Extract Attenuated the Myocardial Injury *In-vivo*

**FIGURE 4:**
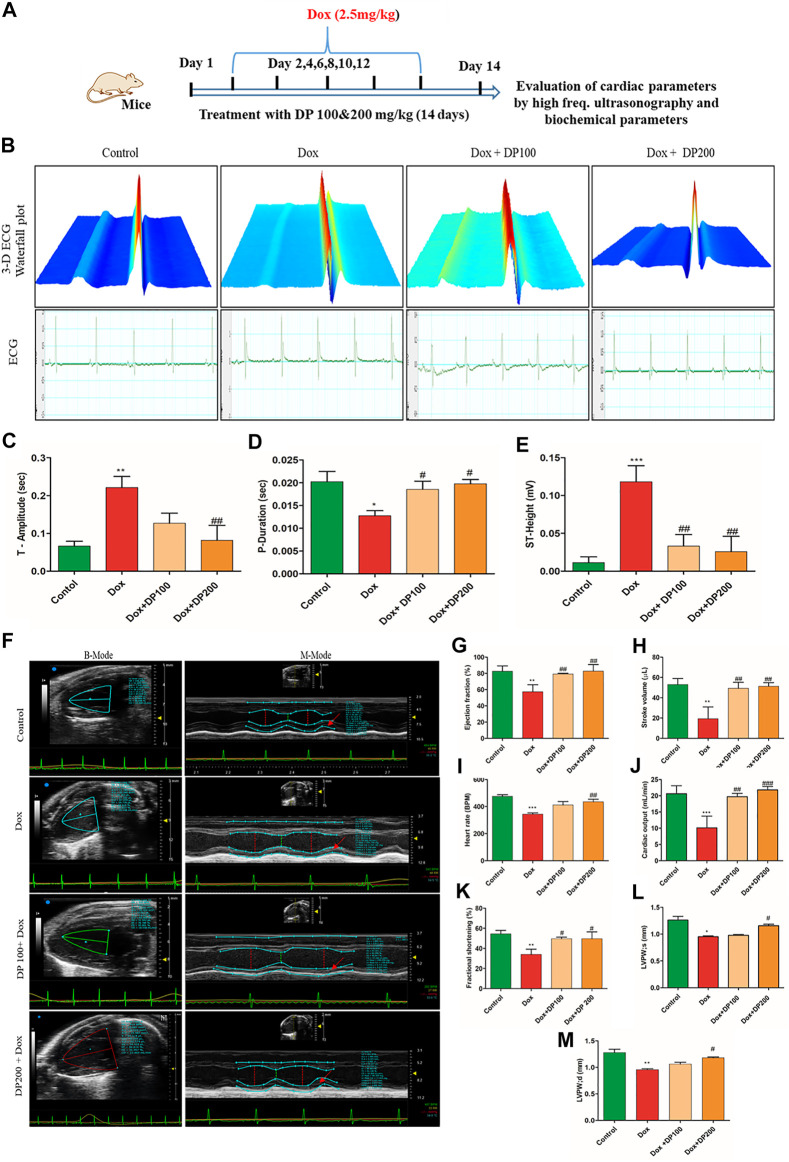
Effect of DP extracts on the electrocardiogram. **(A)** Representative study design for cardioprotective model. **(B)** 3-D representative images of the electrocardiogram with elevated T-wave (I) Control (II) Dox control (III) Dox + DP100 and (IV) Dox + DP 200 **(C)** P-Duration **(D)** T- Amplitude **(E)** ST- Height, where *n* = 5. Effect of DP extract on *in-vivo* cardiac parameters by Vevo Lazer X 3100. **(F)** Representative images of M-Mode (I) control (II) Dox control (III) Dox + DP 100and (IV) Dox + DP200. **(G)** Ejection fraction. **(H)** Stroke volume. **(I)** Heart rate. **(J)** Cardiac output **(K)** Fractional shortening **(L)** left ventricular posterior wall thickness (systole) **(M)** left ventricular posterior wall thickness (diastole), where *n* = 4. The data were analyzed by One Way ANOVA using Graph Pad Prism. ***p < 0.001, **p < 0.01, *p < 0.05 represents normal control vs disease control (Dox). ^#^ *p* < 0.05 ^##^ *p* < 0.01 and ^###^ *p* < 0.001 represents disease control (Dox) vs treatment groups.

The cardioprotective effect of the bioactive fraction was evaluated in a Dox-induced cardiotoxicity mice model (Figure 4A). Electrocardiogram measurements were performed in the various groups to assess the impact of the bioactive DP. A significant decrease in the heart rate (bradycardia) was evidenced in the Dox treated group (394.3 ± 27.43) in comparison to the control mice (499.04 ± 40.49). However, the bioactive DP hydroalcoholic extract treated groups (382.7 ± 59.4 & 447.04 ± 74.7) have significantly improved heart rate than the Dox group. The heart rate of the DP hydroalcoholic extract-treated group was near to the control mice. In Figure 4B representative 3D and ECG images of T-wave elevation were observed in the Dox group. T-wave and ST height elevation was found in the disease control, where it was normalized in the treatment groups, as shown in Figures 4C–E.

### Bioactive DP Hydroalcoholic Extract Alleviates Functional Heart Parameters

Figure 4F shows the representative M-mode images obtained from the parasternal long-axis view images by using a high-frequency ultrasonography-based electrocardiograph imaging system. The left ventricular (LV) posterior wall thickness, ejection fraction, fractional shortening, cardiac output, and stroke volume of the Dox treated group was decreased compared to the control group (Figures 4D–M). Simultaneously, the bioactive DP hydroalcoholic extract-treated groups have restored cardiac parameters and were found to be near the control group.

### Bioactive DP Hydroalcoholic Extract Alleviates Functional Heart Parameters

**FIGURE 5:**
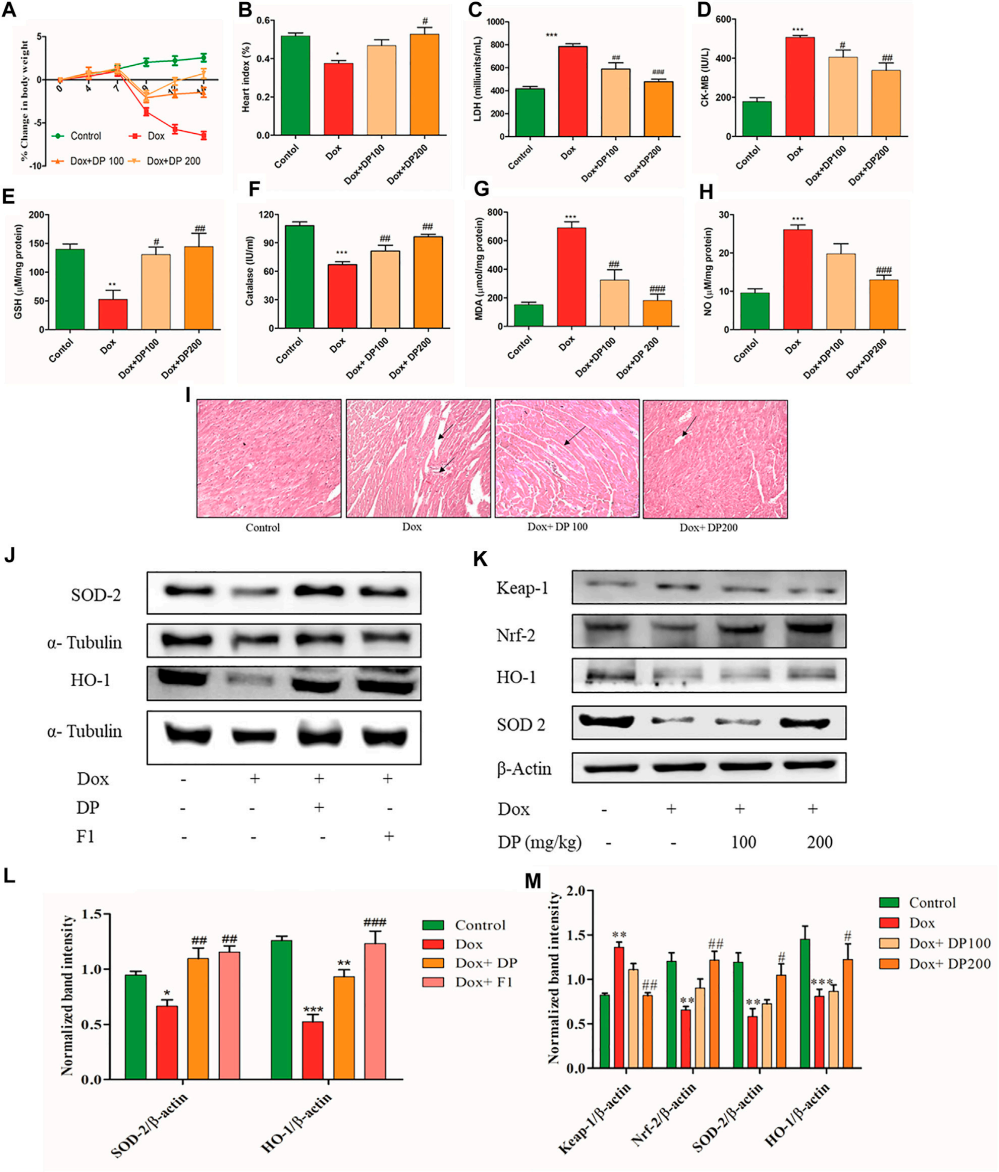
Effect of DP extract on **(A)** percentage change in body weight **(B)** Heart index **(C)** LDH **(D)** CK-MB **(E)** GSH **(F)** catalase **(G)** MDA and **(H)** Nitric oxide, where *n* = 5. **(I)** Histopathology H&E staining of cardiac tissue. **(J)** *In-vitro* western blot analysis of protein levels of SOD-2 and HO-1 in H9c2 cell line **(K)** *In-vivo* western blot analysis of SOD-2, Keap-1, Nrf-2, and HO-1. **(L)** Quantitative analysis of relative protein levels of SOD-2 and HO-1. **(M)** Quantitative analysis of relative protein levels of KEAP-1, Nrf-2 SOD-2, and HO-1 expression respectively. The data were analyzed by One Way ANOVA and Two Way ANOVA using Graph Pad Prism. ***p < 0.001, **p < 0.01, *p < 0.05 represents normal control vs disease control (Dox). ^#^ *p* < 0.05 ^##^ *p* < 0.01 and ^###^ *p* < 0.001 represents disease control (Dox) vs treatment groups (*n* = 3).

The mice’s body weights at the end of the study indicated that the disease control group had shown remarkably reduced body weights compared to the control group. The DP (100 and 200 mg/kg) pre-treated groups had maintained the body weight to its initial values, as shown in Figure 5A. The heat index (HI), a ratio of heart weight to body weight, was lower in the Dox treated group when compared to the control group. The DP pre-treated group has values almost near the normal saline group, as shown in Figure 5B. Further, key biomarker enzymes for tissue damage in the heart, i.e., Creatine Kinase isoenzyme-MB (CK-MB) and lactate dehydrogenase (LDH) levels were analyzed. The Dox alone treated mice group has shown an increase in both the enzymes’ levels than the control group. This reveals that the Dox treated group elevated oxidative stress where lipid peroxidation causes tissue damage followed by enzymatic heart levels (LDH and CK-MB) increase.

Meanwhile, bioactive DP group mice have significantly reduced the levels compared to the control group, as shown in Figures 5C,D. This indicates the protective role of the bioactive DP in alleviating Dox-induced cardiac tissue damage. Also, the levels of anti-oxidant key regulators GSH and catalase were remarkably increased in the treatment groups, as shown in Figures 5E,F. The tissue MDA and nitric oxide (NO) levels were significantly elevated in the Dox treated groups compared to the control group. Bioactive DP treated groups have decreased the levels in a dose-dependent manner, as shown in Figures 5G,H. Histopathological study of the heart tissue in normal control mice exhibited apparent integrity of the myocardial cell membrane with no evident inflammatory cell infiltration, as shown in Figure 5I. The Dox treated mice group showed necrosis and filtration of the lymphocytes, macrophages, and inflammatory cells along with the vacuolization. In some of the myocytes, the myofibrils were disorganized and evident with clear space, indicating intracellular edema. Pre-treatment of the bioactive DP (100 and 200 mg/kg) prevented the entry of the inflammatory cells and vacuole changes caused by the Dox treatment.

### Bioactive Fractions Attenuate Dox-Induced Cardiotoxicity by Activating Nrf-2 Expression

Nrf-2, a key anti-oxidant transcription factor, plays a vital role in maintaining redox homeostasis under oxidative stress. Figure 5J showed treatment with DP alleviated the Dox-induced cardiotoxicity by activating the Nrf-2 expression where the subsequent downstream protein levels were increased *in-vitro*. We found that Keap-1 protein levels were decreased, and Nrf-2 levels were raised in the treatment group in a dose-dependent manner compared with Dox control *in-vivo* (Figure 5K). The quantitative analysis SOD-2 and HO-1 protein levels were increased in the treatment group compared with the Dox control group in both *in-vitro* and *in-vivo* shown in Figures 5L,M. Overall, results indicated that the bioactive fraction attenuated the Dox-induced cardiotoxicity by the Nrf-2 pathway.

#### LC-MS (LC-QTOF-ESI-MS) Analysis

**FIGURE 6:**
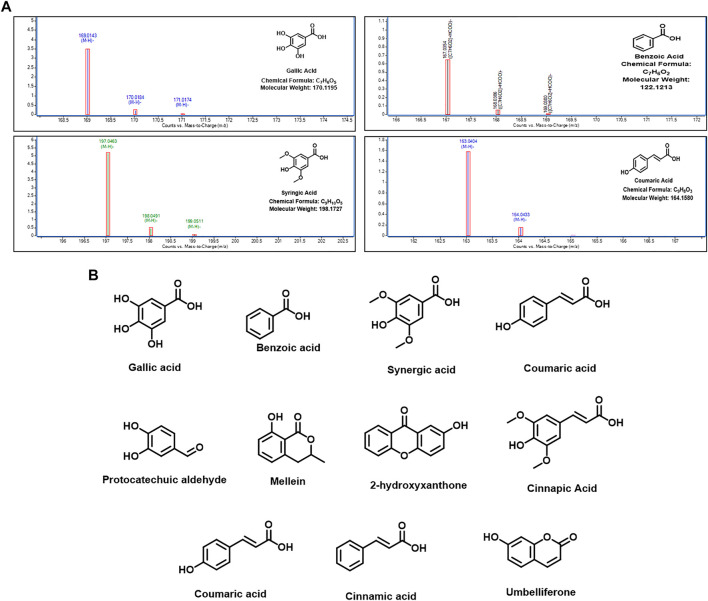
Identified metabolite hits in the hydroalcoholic extract and its fraction F1 in the LC-QTOF-ESI-MS analysis. **(A)** Major metabolites present in both DP and F1. **(B)** Chemical compound structures present in both positive and negative mode in the LC-QTOF-ESI-MS.

**FIGURE 7:**
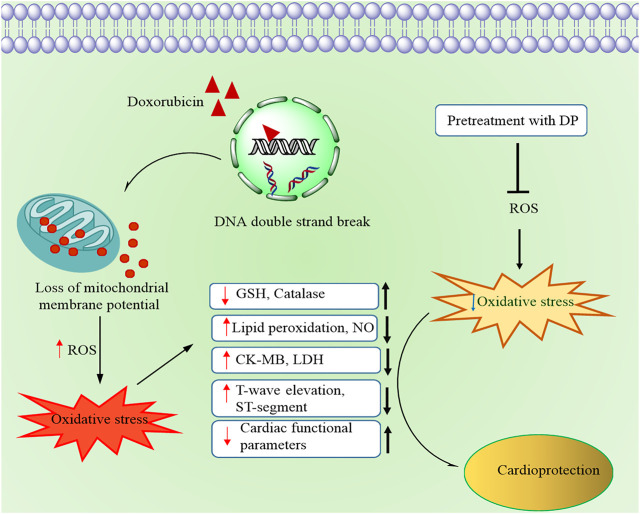
Schematic representation of the proposed cardioprotective mechanism by the hydroalcoholic extract of DP

Hydroalcoholic extract of DP and the fraction F1 were subjected to characterize the lead compounds responsible for the biological activity. Mass accuracy measurements were observed to be within the limit of ±10 PPM. The obtained mass of the molecules/metabolites present in the sample matches with the available literature, as shown in Tables 1–4. Chromatogram for the hydroalcoholic extract of DP and its fraction F1 have been portrayed in Figure 6A. The samples were screened for identification hits based on screening scores and the targeted identification of plant metabolites using a find-by-formula algorithm. However, it may be concluded that most of the molecules have been observed for the formate and acetate adduct form. Polyphenolic compounds gallic acid, benzoic acid, syringic acid, coumaric acid, protocatechuic aldehyde, mellein, 2-hydroxy xanthone, sinapic acid are active chemical constituents in the hydroalcoholic extract and its fraction F1 as detected in the LC-QTOF-ESI-MS, negative mode (Tables 1, 3). However, coumaric acid, cinnamic acid, and umbelliferone are the common active chemical constituents in the hydroalcoholic extract and fraction F1 in the LC-QTOF-ESI-MS analysis in the positive mode, as portrayed in Tables 2, 4 and represented and the structures illustrated in Figure 6B. The proposed cardio protective mechanism of HC has been represented in the Figure 7.

**TABLE 1:** QTOF-ESI-MS data of identified plant metabolites from hydroalcoholic extract of DP in negative mode [M−H]^−^ with the possible molecular formula.

S. No.	Retention time	Name	Molecular formula	Mean measured mass (Da) m/z	Mass (Da)	Diff (Tgt, ppm)	Reference
1	1.035	Gallic acid	C_7_ H_6_ O_5_	169.01	170.12	0.57	Uddin et al. (2020)
2	6.33	Benzoic acid	C_7_ H_6_ O_2_	167.03	122.12	3.47	Yang et al. (2018)
3	6.364	Syringic acid	C_9_ H_10_ O_5_	197.04	198.17	3.68	Ding et al. (2017)
4	6.081	Coumaric acid	C_9_ H_8_ O_3_	163.04	164.16	1.21	Rafiee et al. (2020)
5	6.364	Protocatechuic aldehyde	C_7_ H_6_ O_3_	197.04	138.12	5.28	Jiang et al. (2019)
6	5.914	Paeonol	C_9_ H_10_ O_3_	165.05	166.17	2.53	Ding et al. (2016)
7	6.447	Hesperetin	C_16_ H_14_ O_6_	347.07	302.27	6.71	
8	7.063	Mellein	C_10_ H_10_ O_3_	237.07	178.18	0.83	
9	6.214	Scopoletin	C_10_ H_8_ O_4_	237.04	192.16	4.86	
10	0.636	2-Hydroxyxanthone	C_13_ H_8_ O_3_	257.04	212.20	−3.27	
11	6.663	Herniarin	C_10_ H_8_ O_3_	235.06	176.16	3.61	
12	6.397	Sinapic acid	C_11_ H_12_ O_5_	223.06	224.21	1.95	Bin Jardan et al. (2020)
13	6.43	Naringenin	C_15_ H_12_ O_5_	317.06	272.26	5.75	

**TABLE 2:** QTOF-ESI-MS data of identified plant metabolites from hydroalcoholic extract of DP in positive mode [M+H] ^+^ with possible molecular formula.

Peak no.	Retention time	Name	Molecular formula	Mean measured mass (Da) m/z	Mass (Da)	Diff (Tgt, ppm)	
1	21.107	Coumaric acid	C_9_ H_8_ O_3_	165.05	192.17	1.45	
2	0.995	Cinnamic acid	C_9_ H_8_ O_2_	149.05	148.16	2.38	
3	5.857	Herniarin	C_10_ H_8_ O_3_	177.05	176.16	−2.91	
4	16.279	Umbelliferone	C_9_ H_6_ O_3_	163.03	162.12	2.49	Luo et al. (2018)

**TABLE 3:** QTOF-ESI-MS data of identified plant metabolites from fraction F1 in negative mode [M−H]^−^ with a possible molecular formula.

S. NO.	Retention time	Name	Molecular formula	Mean measured mass (Da) m/z	Mass (Da)	Diff (Tgt, ppm)
1	6.958	Benzoic acid	C_7_ H_6_ O_2_	167.03	122.12	4.43
2	1.047	Gallic acid	C_7_ H_6_ O_5_	169.01	170.12	3.23
3	7.308	Ellagic acid	C_14_ H_6_ O_8_	301.00	302.0077	4.83
4	7.008	Syringic acid	C_9_ H_10_ O_5_	197.04	198.21	4.31
5	3.046	Protocatechuic aldehyde	C_7_ H_6_ O_3_	137.18	138.12	3.18
6	6.709	Coumaric acid	C_9_ H_8_ O_3_	163.04	164.0481	4.4
7	8.241	Dihydroquercetin	C_15_ H_12_ O_7_	303.05	304.25	4.74
8	7.358	Epi-catechin-3-gallate (ECG)	C_22_ H_18_ O_10_	441.08	442.40	4.76
9	10.672	Hispidulin	C_16_ H_12_ O_6_	299.05	462.40	5.29
10	0.831	2-Hydroxyxanthone	C_13_ H_8_ O_3_	257.04	212.20	−4.78
11	1.83	2,3-Dihydroxybenzoic acid	C_7_ H_6_ O_4_	153.01	154.12	3.73
12	10.838	Cirsilineol	C_18_ H_16_ O_7_	343.08	344.30	3.56
13	1.813	Catechol	C_6_ H_6_ O_2_	109.03	110.10	3.98
14	11.721	Formononetin	C_16_ H_12_ O_4_	327.08	268.26	5.16
15	6.909	Hydroxycaffeic acid	C_9_ H_28_ O_5_	241.03	196.16	2.96
16	10.072	Cirsimaritin	C_17_ H_14_ O_6_	359.07	314.29	5.34
17	10.072	Rosmarinic acid	C_18_ H_16_ O_8_	359.07	360.31	4.66
18	6.942	Umbelliferone	C_9_ H_6_ O_3_	207.03	162.14	5.62
19	0.648	5,6-Dihydroxy-7,8,3′,4′-tetramethoxyflavone	C_19_ H_18_ O_8_	419.09	406.34	2.27
20	6.692	Eriodictyol 7-O-glucoside	C_21_ H_22_ O_11_	509.13	450.39	5.96
21	10.672	Dihydroxyflavone	C_15_ H1_0_ O_4_	299.05	254.24	5.83
22	12.387	Glycyrin	C_22_ H_22_ O_6_	427.14	382.40	3.03
23	6.709	Citropten	C_11_ H_10_ O_4_	265.07	206.19	3.97
24	7.341	Caffeic acid	C_9_ H_8_ O_4_	225.04	180.16	3.42
25	7.441	Sinapic acid	C_11_ H_12_ O_5_	223.06	224.06	4
26	7.441	Mellein	C_10_ H_10_ O_3_	223.06	178.18	5.03

**TABLE 4:** QTOF-ESI-MS data of identified plant metabolites from fraction F1 in positive mode [M+H]^+^ with the possible molecular formula.

Peak no.	Retention time	Name	Molecular formula	Mean measured mass (Da) m/z	Mass (Da)	Diff (Tgt, ppm)
1	21.107	Coumaric acid	C_9_ H_8_ O_3_	165.05	192.17	1.45
2	5.857	Herniarin	C_10_ H_8_ O_3_	177.05	176.16	−2.91
3	16.279	Umbelliferone	C_9_ H_6_ O_3_	163.03	162.12	2.49

## Discussion and Conclusion

Recent progress in cancer therapy has increased patients’ life expectancy and elevated the cardiac complications in them. Doxorubicin (Dox) is a widely known chemotherapeutic drug, which induces oxidative stress, leading to free radicals, subsequently causing oxidative damage to the cardiomyocytes (Pugazhendhi et al., 2018). Dox-induced cardiotoxicity model has been successfully demonstrated in various cultured cell lines (Chan et al., 1996), isolated heart cultures, whole animal models, and humans (Singal et al., 1987). Identification and evaluation of safer bioactive molecules/fractions, which help alleviate the existing chemotherapeutic drugs’ cardiotoxic effect, would benefit cancer patients.

*Dillenia pentagyna* Roxb. is a nutritionally and medicinally valued plant distributed widely in South Asian countries. The fruits and bark of the plant were used for the treatment of various ailments. In the current study, we intend to evaluate the anti-oxidant role in alleviating the Dox-induced cardiotoxicity both *in-vitro* and *in-vivo*. The preliminary anti-oxidant tests suggested that the *Dillenia* pentagyna Roxb’s hydroalcoholic extract had high anti-oxidant chemical compounds. Therefore, the bioactive DP was further subjected to fractionation to obtain a purified bioactive fraction (Fraction-1).

However, anti-oxidant chemical tests indicated that both the DP and Fraction-1 have an equivalent amount of anti-oxidant compounds, and fraction F1 has high phenolic content than the crude DP. The pretreatment with DP and F1 has shown the increased cell viability in H9c2 cells indicating the protective activity. Multiple pieces of evidence revealed that the reactive oxygen species (ROS) has a direct implication in the pathogenesis of Dox-induced cardiotoxicity (Hwang et al., 2008; Sun et al., 2015). *In-vitro* DCFDA and Mitosox staining revealed that the Dox treated cardiomyocytes (H9c2) have high levels of reactive oxygen species, which is evident by the fluorescence signal in both the flow cytometric and microscopic analysis. Simultaneously, the cells treated with the bioactive DP have shown a less amount of fluorescence, indicating the reduced ROS. The previous reports suggest that anti-oxidants can modulate the anticancer drug efficacy in cancer treatment (Conklin, 2004). However, the treatment with DP and F1 and Dox on the cancer cell line did not compromise the anticancer activity of Dox in the cancer cell line.

When evaluated by the ECG, the cardioprotective activity of the bioactive DP evidenced normalization of ECG changes, most notably ST height and T-wave elevation. An altered ECG was due to the propagation of lipid peroxidation in the Dox treated mice group as the bioactive fractions reduced lipid peroxidation, which could be responsible for the normalized T-wave elevation and ST-height. Electrocardiographic (ECG) imaging is a non-invasive method to study the various cardiac parameters. In the current study, we have used the electrocardiography-based imaging technique to study the bioactive DP’s cardioprotective effect in Dox-induced cardiotoxicity models. The application of such techniques provides reliable information about the cardiac profile. Dox causes a decrease in left ventricular wall thickness (Prathumsap et al., 2020), cardiac functional parameters ejection fraction, fractional shortening, stroke volume, cardiac output, and heart rate. Our findings of increased cardiac functional parameters indicated that DP had reduced the toxic effects of Dox-induced cardiotoxicity. In our study, *in vivo* anti-oxidant markers like GSH and catalase were also elevated, indicating the endogenous anti-oxidant defense system. Cardiac tissue biomarkers like Creatine kinase isoenzyme-MB (CKMB), Lactate dehydrogenase (LDH) were released when damage to the cardiomyocytes. We found that the Dox treatment has increased their levels in the Dox group, whereas the pre-treatment significantly decreased the levels with bioactive DP, indicating the attenuation of the cardiac damage by Dox. The same was further supported by estimating tissue-based lipid peroxidation end product MDA and the nitrite levels. We found that the levels were significantly decreased, indicating that treatment with DP inhibited lipid peroxidation.

Nuclear factor (erythroid-derived 2)-like 2 (Nrf-2), a redox-sensitive transcription factor regulating various cellular responses to electrophilic/oxidative stress. It has previously been revealed the role of Nrf-2 in the detoxification process in cardiac cells (Howden, 2013). Therefore, growing evidence suggested that Nrf-2 may target Dox-induced cardiotoxicity treatment (Tomlinson et al., 2019; Zhang et al., 2020). Our study results indicated that treatment with the bioactive DP increased the expression of Nfr-2 a critical anti-oxidant regulator of oxidative stress, by decreasing its negative regulator Keap-1 expression. The downstream proteins expression like SOD-2 and HO-1 were elevated, indicating the rise of anti-oxidant enzyme expression that counteracts the produced ROS in H9c2 cell line and *in vivo* anti-oxidant enzyme expression that neutralizes the produced ROS in H9c2 cell line and *in-vivo* evident from the western blot analysis. This proves that the bioactive DP ameliorated Dox-induced oxidative stress.

It is evident from this study that identified metabolite hits gallic acid, benzoic acid, syringic acid, coumaric acid, protocatechuic aldehyde, mellein, 2-hydroxy xanthone, sinapic acid, coumaric acid, cinnamic acid, and umbelliferone are phenolic containing chemical constituents present in hydroalcoholic extract DP and its F1 fraction in the LC-QTOF-ESI-MS. Therefore, it may be concluded that hydroalcoholic extract and its fraction F1 are enriched with polyphenols and have shown good cardioprotective activity in the preclinical screening model.

Taken together, our data showed convincing evidence that pre-treatment with DP and F1 alleviated the cardiotoxicity *in-vitro. In-vivo* mice model also indicated that the DP caused no significant effect to the body weights, meanwhile restoring the cardiac parameters nearly equal to the normal control group. Pretreatment with DP attenuated the Dox-induced cardiotoxicity by reducing the oxidative stress, and the Nrf-2 pathway mediated the cardioprotection. Further *in-vivo* studies are required to understand the role of fraction F1, and extensive studies have to be performed to know the exact molecules involved in the protective effect of the DP against Dox-induced cardiotoxicity.

## Data Availability

The original contributions presented in the study are included in the article/Supplementary Material, further inquiries can be directed to the corresponding authors.

## Abbreviations

DPPH, 2,2-diphenyl-1-picryl-hydrazyl; ABTS, 2,2′-azino-bis(3-ethylbenzo) thiazoline-6-sulfonic acid; TPC, Total phenolic acid; RIPA buffer; MTT, 3- (4,5-dimethylthiazol-2-yl) -2,5-diphenyl tetrazolium bromide; FRAP, ferric reducing antioxidant power; CM-H2DCFDA, chloromethyl derivative of 2′, 7′ –dichlorofluorescein (DCF); ROS, Reactive oxygen species; FACS, fluorescence-activated cell sorting; DMSO, dimethylsulfoxide; ESI, electronspray ionization; ATCC, American type culture collection; NCCS, National center for cell science; SOD-2, superoxide dismutase; Keap-1, Kelch-like ECH-associated protein 1; Nrf-2, nuclear factor erythroid 2-related factor 2; HO-1, hemeoxygenase; RSA, radical scavenging assay; BSA, bovine serum albumin; LCMS-QTOF, Liquid chromatograpy-quadrapole time of flight; HFUS- high frequency ultrasonography.
