# Supplementary material for: Polyphenolic-Rich Compounds From Dillenia pentagyna (Roxb.) Attenuates the Doxorubicin-Induced Cardiotoxicity: A High-Frequency Ultrasonography Assisted Approach
Source: Front Pharmacol. 2021 May 17;12:624706. doi: 10.3389/fphar.2021.624706 (PMC8166202; doi:10.3389/fphar.2021.624706)
Supplement: Supplementary file 1 [file table1.docx]

**List of figures:**

**Fig. 1:** Schematic representation of the proposed study design cardioprotective mechanism by the hydroalcoholic extract of DP.

**Fig. 2**: IC_50_ values of the bioactive fractions in various antioxidant assays. A) ABTS and B) DPPH assay compared with ascorbic acid. C) FRAP assay (equivalent ascorbic acid /gm extract) D) Total phenolic content (Gallic acid equivalent/gm extract). E, F) Cell viability of cardiomyocytes (H9c2) pre-treatment with DP and Fraction F1 along with Dox. Values were expressed as Mean ± SEM (n=3). The data were analyzed by One Way ANOVA using Graph Pad Prism. ***P <0.001, and **P <0.01represents Control vs Dox control. ^##^P<0.01 and ^###^P<0.001 represents Dox control vs Treatment groups.

**Fig. 3:** *In-vitro* evaluation of the cardioprotective effect of the DP and fraction F1. A) Quantitative analysis of ROS by DCFDA using flow cytometer. B) Histogram overlay of DCFDA shift C) Representative brightfield and fluorescent images of the DCFDA stained cells. D) Mean fluorescence intensity of DCFDA. E) Representative superoxide radical generation using Mitosox staining. F) Quantitative measurement of mean fluorescence intensity (MFI) of Mitosox red. G) Troponin I immunoflourscence indicating vacuolization. The data were analyzed by One Way ANOVA using Graph Pad Prism. ***P <0.001, and **P <0.01represents Control vs Dox control. ^##^P<0.01 and ^###^P<0.001 represents Dox control vs Treatment groups (n=3).

**Fig. 4:** Effect of DP extracts on the electrocardiogram. A) Representative study design for cardioprotective model. B) 3-D representative images of the electrocardiogram with elevated T- wave (I) Control (II) Dox control (III) Dox+DP100 and (IV) Dox+ DP 200 C) P-Duration D) T- Amplitude E) ST- Height, where n=5. Effect of DP extract on *in-vivo* cardiac parameters by Vevo Lazer X 3100. F) Representative images of M-Mode (I) control (II) Dox control (III) Dox + DP 100and (IV) Dox + DP200. G) Ejection fraction H) Stroke volume I) Heart rate J) Cardiac output K) Fractional shortening L) left ventricular posterior wall thickness (systole) M) left ventricular posterior wall thickness (diastole), where n=4. The data were analysed by One Way ANOVA using Graph Pad Prism. ***p<0.001, **p<0.01, *p <0.05 represents normal control vs disease control (Dox). ^#^p<0.05 ^##^p<0.01 and ^###^p<0.001 represents disease control (Dox) vs treatment groups.

**Fig. 5:** Effect of DP extract on A) percentage change in body weight B) Heart index C) LDH D) CK-MB E) GSH F) Catalase G) MDA and H) Nitric oxide, where n=5. I) Histopathology H&E staining of cardiac tissue. J) *In-vitro* western blot analysis of protein levels of SOD-2 and HO-1 in H9c2 cell line K) *In-vivo* western blot analysis of SOD-2, Keap-1, Nrf-2, and HO-1. L) Quantitative analysis of relative protein levels of SOD-2 and HO-1. M) Quantitative analysis of relative protein levels of KEAP-1, Nrf-2 SOD-2, and HO-1 expression respectively. The data were analyzed by One Way ANOVA and Two Way ANOVA using Graph Pad Prism. ***p<0.001, **p <0.01, *p<0.05 represents normal control vs disease control (Dox). ^#^p<0.05 ^##^p<0.01 and ^###^p<0.001 represents disease control (Dox) vs treatment groups (n=3).

**Fig. 6**: Identified metabolite hits in the hydroalcoholic extract and its fraction F1 in the LC-QTOF-ESI-MS analysis. A) Major metabolites present in both DP and F1. B) Chemical compound structures present in both positive and negative mode in the LC-QTOF-ESI-MS.

**Fig. :** Schematic representation of the proposed cardioprotective mechanism by the hydroalcoholic extract of DP.

**Supporting Data**

**Supporting fig. 1:** *In-vitro* antioxidant assays. ABTS, DPPH and FRAP assays for all the extracts of *Dillenia pentagyna*.

**Supporting** **fig. 2:** A) Flash chromatogram of the hydroalcoholic fractions of DP. B) Gradient fractionation method.

**Supporting fig. 3:** Effect of DP and fraction F1 on cell viability of H9c2 cell line A) Percentage cell viability of DP alone on H9c2 cells. B) Percentage cell viability of fraction F1 alone on H9c2 cells. Effect of DP and fraction F1 on Dox anticancer activity on colon cancer cell line (HCT-116) C) Percentage cell viability of Dox along with the DP. D) Percentage cell viability of Dox along with the F1. The data were analysed by One Way ANOVA using Graph Pad Prism.***p <0.001 represents normal control vs disease control (Dox). No significant (ns) represents disease control (Dox) vs treatment groups (Dox+DP/F1) (n=3).
