# Supplementary figures and images for: Polyphenolic-Rich Compounds From Dillenia pentagyna (Roxb.) Attenuates the Doxorubicin-Induced Cardiotoxicity: A High-Frequency Ultrasonography Assisted Approach
Source: Front Pharmacol. 2021 May 17;12:624706. doi: 10.3389/fphar.2021.624706 (PMC8166202; doi:10.3389/fphar.2021.624706)

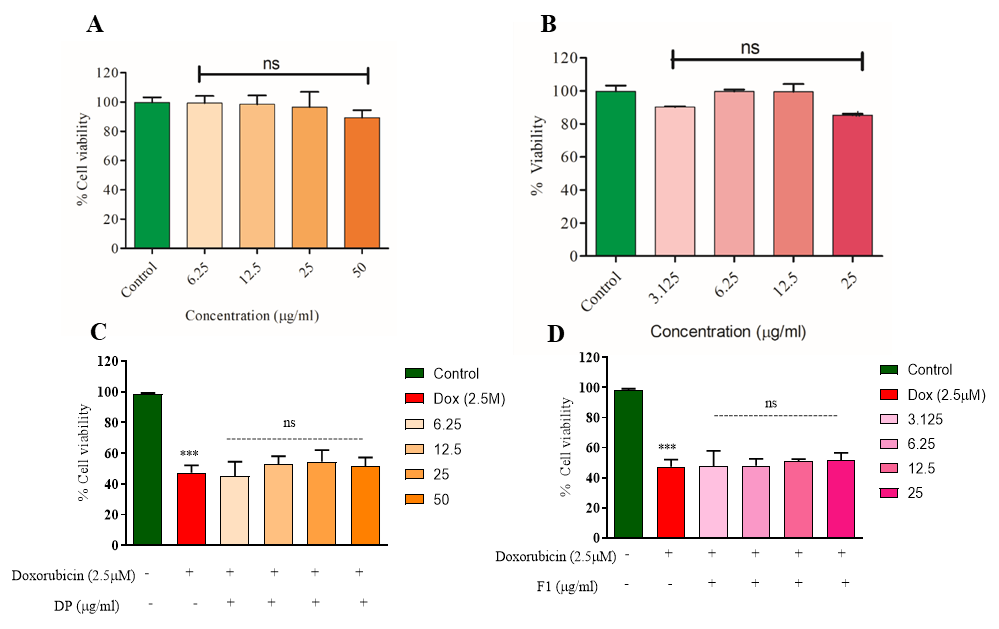

Supplement: Supplementary file 2 [file image3.tif]

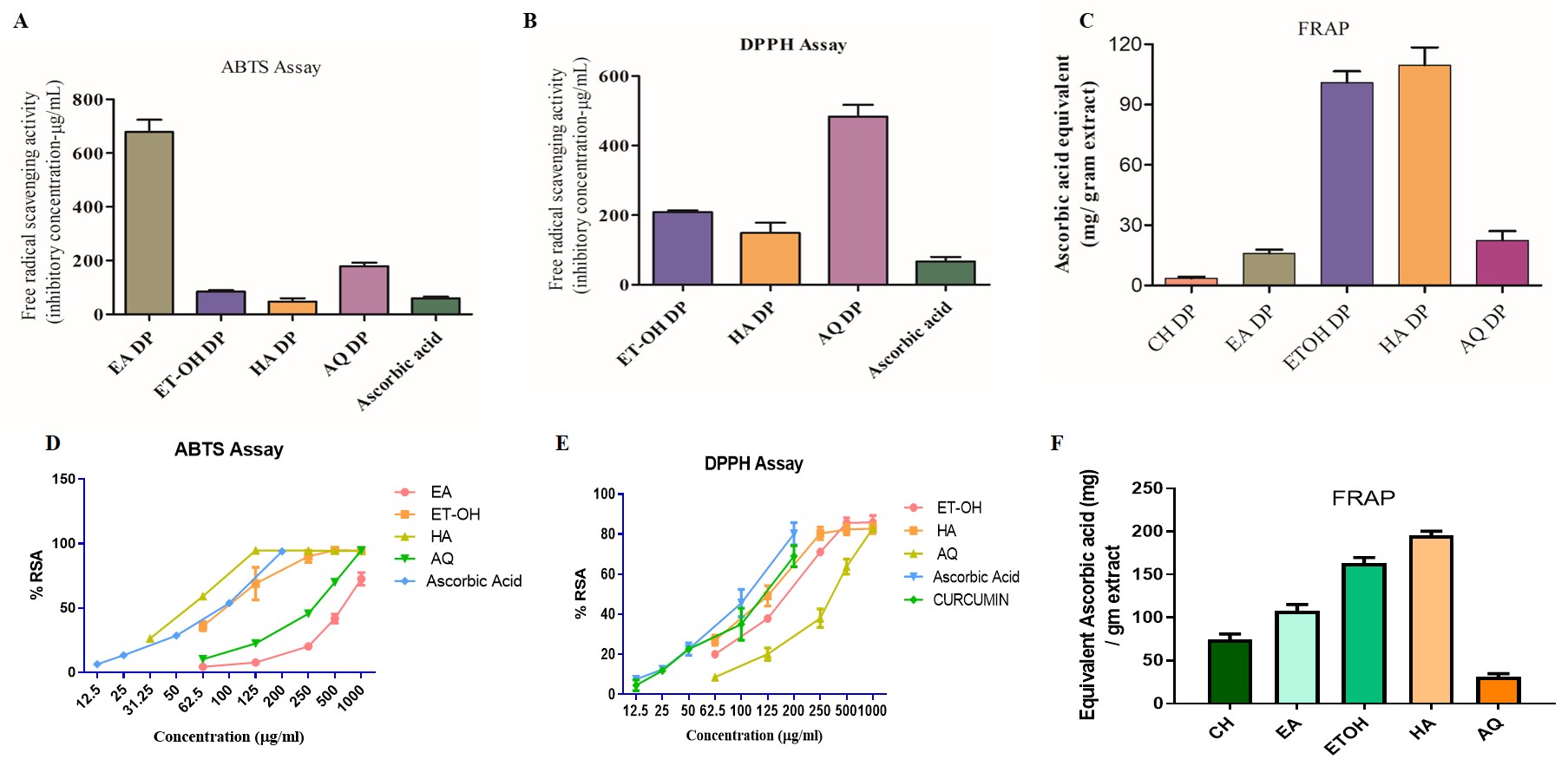

Supplement: Supplementary file 3 [file image1.jpeg]

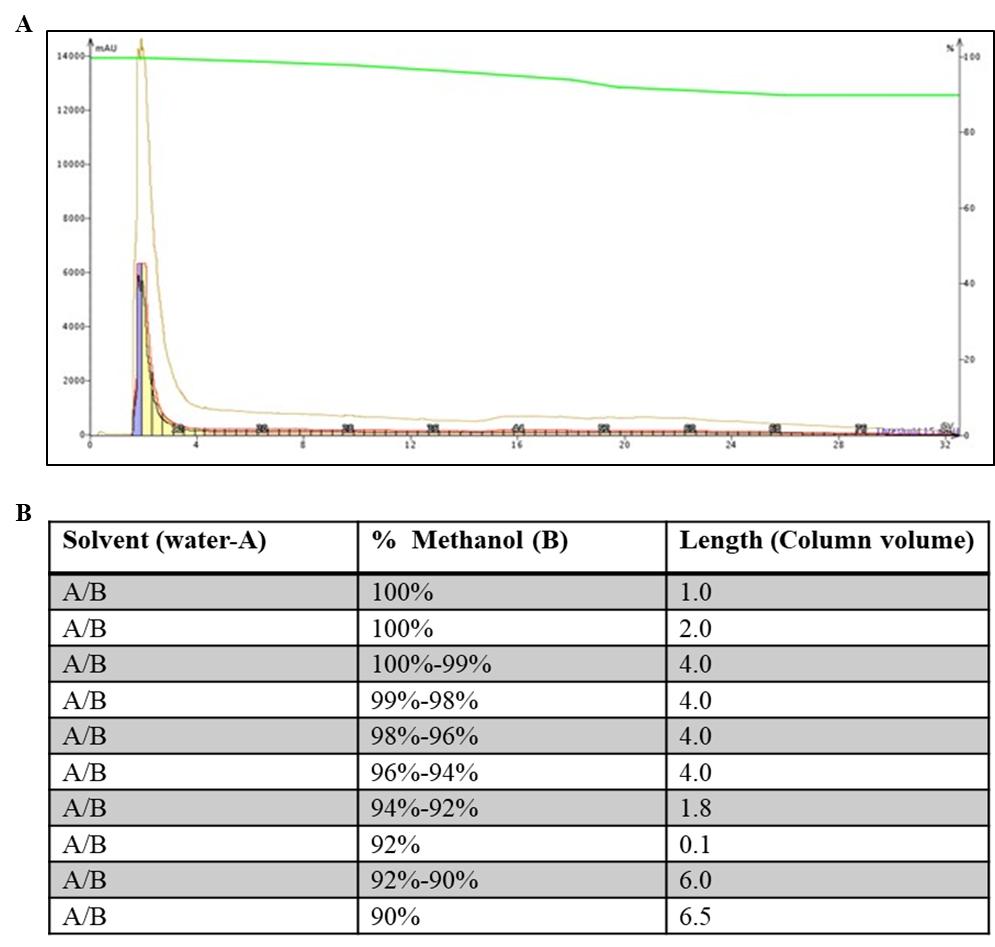

Supplement: Supplementary file 4 [file image2.tif]
